# Incidence and Distinct Features of Immune Checkpoint Inhibitor-Related Myositis From Idiopathic Inflammatory Myositis: A Single-Center Experience With Systematic Literature Review and Meta-Analysis

**DOI:** 10.3389/fimmu.2021.803410

**Published:** 2021-12-06

**Authors:** Naoki Hamada, Ayaka Maeda, Kaoru Takase-Minegishi, Yohei Kirino, Yumiko Sugiyama, Ho Namkoong, Nobuyuki Horita, Ryusuke Yoshimi, Hideaki Nakajima, YCU irAE Working Group, Naoki Hamada

**Affiliations:** ^1^ Department of Stem Cell and Immune Regulation, Yokohama City University Graduate School of Medicine, Yokohama, Japan; ^2^ Department of Infectious Diseases, Keio University School of Medicine, Tokyo, Japan; ^3^ Chemothrapy Center, Yokohama City University Hospital, Yokohama, Japan

**Keywords:** immune checkpoint inhibitor (ICI), irAE, autoimmune, myocarditis, myositis

## Abstract

Immune checkpoint inhibitor (ICI)-related myositis is a rare, potentially fatal condition that warrants further studies. Its incidence, clinical features, and prognosis remain poorly understood. To address these gaps, we conducted a systematic review and meta-analysis to evaluate the risk of myositis associated with ICI for solid tumors by analyzing phase III randomized controlled trials of anti-programmed death-1/ligand-1 (PD-1/PD-L1) and anti-cytotoxic T-lymphocyte antigen-4 (CTLA-4). To complement this analysis with clinical data, we evaluated published ICI case reports along with cases from our institutional registry. This registry comprised 422 patients treated with ICIs alone or in combination from September 2014 to June 2021. The analysis revealed an incidence of ICI-related myositis in 6,838 patients in 18 randomized controlled trials of 0.38% (odds ratio 1.96; 95% confidence interval 1.02–3.75) for patients receiving ICIs compared with controls. Detailed analysis of 88 cases from the literature search and our registry showed that myositis induced by PD-1 inhibitors was more frequent than that induced by anti-CTLA-4 agents, revealing a clinically diverse trend including myasthenia gravis and myocarditis. Importantly, having ptosis at the time of onset was significantly associated with the development of concomitant myocarditis (odds ratio 3.81; 95% CI 1.48–9.83), which is associated with poor prognosis. Regarding treatment, most patients received glucocorticoids, and some received immunosuppressants. Our study revealed the incidence of ICI-mediated myositis and the clinical features of myocarditis, highlighting the need for recognition and early intervention.

## Introduction

Immune checkpoint inhibitors (ICIs) have revolutionized cancer treatment by exerting anti-tumor effects on various types and stages of cancer that cannot be achieved with existing drugs, and numerous clinical trials are underway to expand indications (1). However, treatment response to ICIs is variable in terms of both efficacy and adverse effects (2, 3). Autoimmune reactions to various organs—immune-related adverse events (irAEs)—are observed in patients treated with ICIs (4, 5). The phenotypes of irAEs vary and it is currently impossible to predict how often they will occur, and in which organs. ICI-related myositis is rare but has been reported to be potentially fatal (6).

A review by Kadota et al. (7) reported that myositis occurred in patients receiving anti-programmed death-1 (PD-1) alone, anti-cytotoxic T-lymphocyte antigen-4 (CTLA-4) alone, and with combination therapy. Mean time from ICI initiation to the onset of ICI-related myositis was 4 weeks. Causes of death were myocarditis, respiratory paralysis, and cancer progression. In patients without myocarditis or respiratory muscle paralysis, creatine kinase (CK) levels normalized after ICI discontinuation and administration of immunosuppressive drugs, and the prognosis of myositis was good. The review suggested that the clinical features of ICI-related myositis can be divided into two subsets: new onset of myositis as an irAE, and onset of idiopathic inflammatory myositis (IIM). However, because of its rarity, the incidence, clinical features, and prognosis of ICI-related myositis are still poorly understood.

A systematic review on myocarditis has been published (8), but, to our knowledge, no meta-analysis on this topic has been conducted. Since the 2019 publication of the Kadota et al. review (7) many additional cases have been reported, thus there is the opportunity to clarify some of the questions about this condition. With this aim, we conducted a systematic review on ICI-related myositis. To clarify the detailed clinical features of ICI-related myositis, we searched the literature for case reports together with data from the Yokohama City University ICI registry. Here, we report the incidence, clinical features, and potential predictors of fatal myocarditis in these patients.

## Materials and Methods

### Systematic Review and Meta-Analysis of Randomized Controlled Trials

The systematic review was conducted following the Preferred Reporting Items for Systematic Reviews and Meta-Analyses guidelines (9, 10) and was registered in the University Hospital Medical Information Network Center Clinical Trial Registry (Japan) as UMIN000044960 (11). Institutional Review Board approval and patient informed consent were waived for the systematic review and meta-analysis due to nature of the study.

In the electronic search, we systematically searched PubMed, EMBASE, the Cochrane Central Register of Controlled Trials, and Web of Science Core Collection (up to August 16 2021) for randomized controlled trials (RCTs) reporting the risk of myositis associated with ICIs for the treatment of patients with solid tumors. Search formulae are presented in Supplementary Text 1. Two investigators (AM, KT-M) independently screened candidate articles by checking the title and abstract after uploading the citation list into Endnote X9 software (Thomson Reuters, Philadelphia, PA, USA). Inclusion criteria were as follows: (1) phase III RCT study design; (2) the experimental group was treated with at least one type of ICI with or without other systemic chemotherapy and the control group with non-ICIs; (3) three-arm studies where an ICI was included in at least one arm; (4) patients clinically diagnosed with any solid tumor; and (5) the study included the incidence of myositis. Exclusion criteria were as follows: (1) systematic review or meta-analyses; (2) retrospective analyses; (3) single prospective cohort studies with no control group; (4) ICI two-drug combination therapy; (5) republished research literature; (6) studies with no or insufficient safety results at the time of the literature search; and (7) studies published in languages other than English. Only full-text papers were used for analysis. Disagreements in assessing cases or data were resolved *via* discussion between the two investigators.

Odds ratios (ORs) of any-grade myositis between the ICI treatment arm and non-ICI arm were calculated using a random-effect model meta-analysis. Heterogeneity was assessed using I^2^ statistics and P-values. Heterogeneity was indicated by I^2^ wherein 0% indicated no heterogeneity and 100% indicated the strongest heterogeneity. Statistical analyses were performed using Review Manager 5.3 (Cochrane Community, London, UK). The Cochrane Risk of Bias Tool was used to evaluate the risk of bias for each RCT (12). The quality of the RCTs was independently assessed by two reviewers (AM, KT-M).

### Patients and Design of the Yokohama City University Institutional Database

Consecutive cancer patients (excluding those in clinical trials) who received ICIs (nivolumab, pembrolizumab, ipilimumab, atezolizumab, or durvalumab) for approved cancer types at the collaborative research department of Yokohama City University Hospital between December 2014 and May 2021 were included. The study was approved by the ethics committee of Yokohama City University (A200500004). In this study, we included patients treated with ICI monotherapy or combination therapy. Demographic, clinical, and treatment data were obtained from clinical charts. The study followed the Ethical Guidelines for Epidemiology Research, published by the Japanese Ministry of Health, Labour and Welfare, and had an opt-out strategy.

### Literature Review of Case Reports

We searched the literature for cases of ICI-related myositis using PubMed on September 7, 2021. ‘ICI-related myositis’ was defined according to the previously reported literature review (7). Cases clinically diagnosed as myositis were included in the analysis, but cases with focal myositis, such as orbital myositis, were excluded. We searched PubMed using the following terms: (pembrolizumab OR nivolumab OR ipilimumab) AND (myositis OR myopathy OR myopathies OR dermatomyositis OR polymyositis). Additional related articles were identified through a manual search of the bibliographies of the included studies to ensure that all relevant studies and recent reviews were included.

Data from case reports were divided into two groups: myositis alone, and concomitant myocarditis. Clinical characteristics, the type of ICI, autoantibody status, management, and outcome were evaluated and compared between two groups.

Clinical data were analyzed using both univariate and multivariate analysis. Comparisons of frequencies were made by Fisher’s exact test, or by chi-square test if there were three or more categories for each variable. Continuous variables were compared using Mann-Whitney U test. Logistic regression models were used to identify multivariate predictors of myocarditis, after adjusting for the effects of age and gender. Values of p<0.05 were considered significant. Statistical analyses were performed using software IBM/SPSS Statistics version 22 (Armonk, NY).

## Results

### Incidence and Risk of ICI-Related Myositis in the Systematic Review and Meta-Analysis

**Figure 1:**
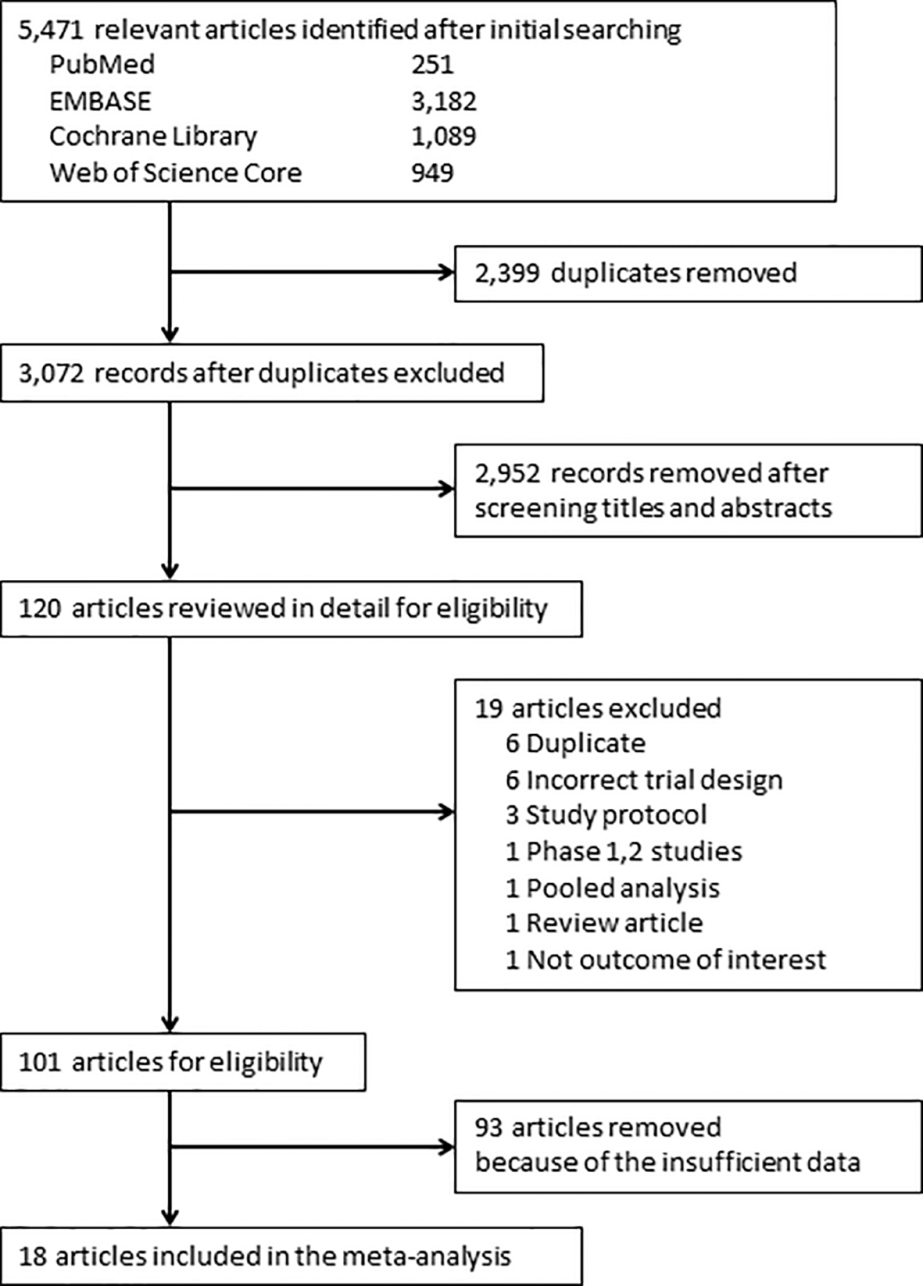
PRISMA flow diagram for the meta-analysis. Study selection process according to the Preferred Reporting Items for Systematic Reviews and Meta-Analyses (PRISMA) guidelines.

Of 5,471 candidate articles, 120 were potentially eligible after abstract and title screening. From these 120, 19 studies were excluded, and 101 articles were reviewed in detail. Studies of 93 RCTs were insufficient to assess the incidence of myositis. Finally, 18 RCTs (13–30) were included in this meta-analysis (**Figure 1**). Characteristics of the included studies are summarized in **Table 1**. Visual inspection of the funnel plot was assessed (**Supplementary Figure 1**). The overall risk of bias for most studies evaluated was also low (**Supplementary Figure 2**). There was no clear risk of publication bias in the included studies.

**Table 1:** Overview of the included studies in the meta-analysis.

First author	Year	Cancer types	Cancer status	Study setting	ICI arm	Non-ICI arm	Number of patients			
							ICI		Non-ICI	
							All	Myositis	All	Myositis
André (12)	2020	MSI-High Colorectal	Advanced	First-line	Pembrolizumab	Chemotherapy	153	1	143	0
Eggermont (13)	2018	Melanoma	Resected stage III	Adjuvant	Pembrolizumab	Placebo	509	1	502	0
Ferris (14)	2016	HNSCC	Recurrent	After platinum-based chemotherapy	Nivolumab	MTX, Docetaxel, or Cetuximab	236	0	111	1
Galsky (15)	2020	Urothelial	Metastatic	First-line	Atezolizumab + platinum-based chemotherapy	Placebo + platinum-based chemotherapy	453	3	390	1
Gutzmer (16)	2020	Melanoma	Advanced	First-line	Atezolizumab	Placebo	230	1	281	0
Kojima (17)	2020	Esophageal	Advanced	Second-line	Pembrolizumab	Chemotherapy	314	1	296	0
Kuruvilla (18)	2021	HL	Relapsed or refractory	Second or later line	Pembrolizumab	Brentuximab	148	1	152	0
Miles (19)	2021	TNBC	Metastatic	First-line	Atezolizumab + PTX	Placebo + PTX	431	1	218	0
Mittendorf (20)	2020	TNBC	Stage II-III	First-line	Atezolizumab	Placebo	164	1	167	0
Moore (21)	2021	Ovarian	Ssage III-IV	Neoadjuvant	Atezolizumab + CBDCA + PTX + Bevacizumab	Placebo + CBDCA + PTX + Bevacizumab	642	4	644	5
Powles (22)	2018	Urothelial	Advanced or metastatic	After platinum-based chemotherapy	Atezolizumab	Vinflunine, PTX, or Docetaxel	459	1	443	0
Powles (23)	2020	Urothelial	Advanced or metastatic	First-line	Avelumab + BSC	BSC alone	344	1	345	0
Reck (24)	2016	SCLC	Extensive-stage	First-line	Ipilimumab + CDDP/CBDCA + VP-16	Placebo + CDDP/CBDCA + VP-16	562	2	561	0
Rini (25)	2019	RCC	Advanced	First-line	Pembrolizumab + Axitinib	Sunitinib	429	2	425	0
Rini (26)	2019	RCC	Metastatic	First-line	Atezolizumab + Bevacizumab	Sunitinib	451	1	446	0
Rudin (27)	2020	SCLC	Stage IV	First-line	Pembrolizumab + EP	Placebo + EP	223	1	223	0
Schmid (28)	2020	TNBC	Stage II-III	First-line	Pembrolizumab + chemotherapy	Placebo + Chemotherapy	781	3	389	0
Winer (29)	2021	TNBC	Metastatic	Second or later line	Pembrolizumab	Chemotherapy	309	1	292	0

MSI-H, microsatellite-instability–high; NSCLC, non-small cell lung cancer; HNSCC, head-and-neck squamous cell cancer; TNBC, triple-negative breast cancer; SCLC, small cell lung cancer; RCC, renal cell carcinoma; ICI, immune checkpoint inhibitor; PTX, paclitaxel; CBDCA, carboplatin; BSC, best supportive care; CDDP, cisplatin; VP-16, etoposide; EP, etoposide and platinum; MTX, methotrexate.

**Figure 2:**
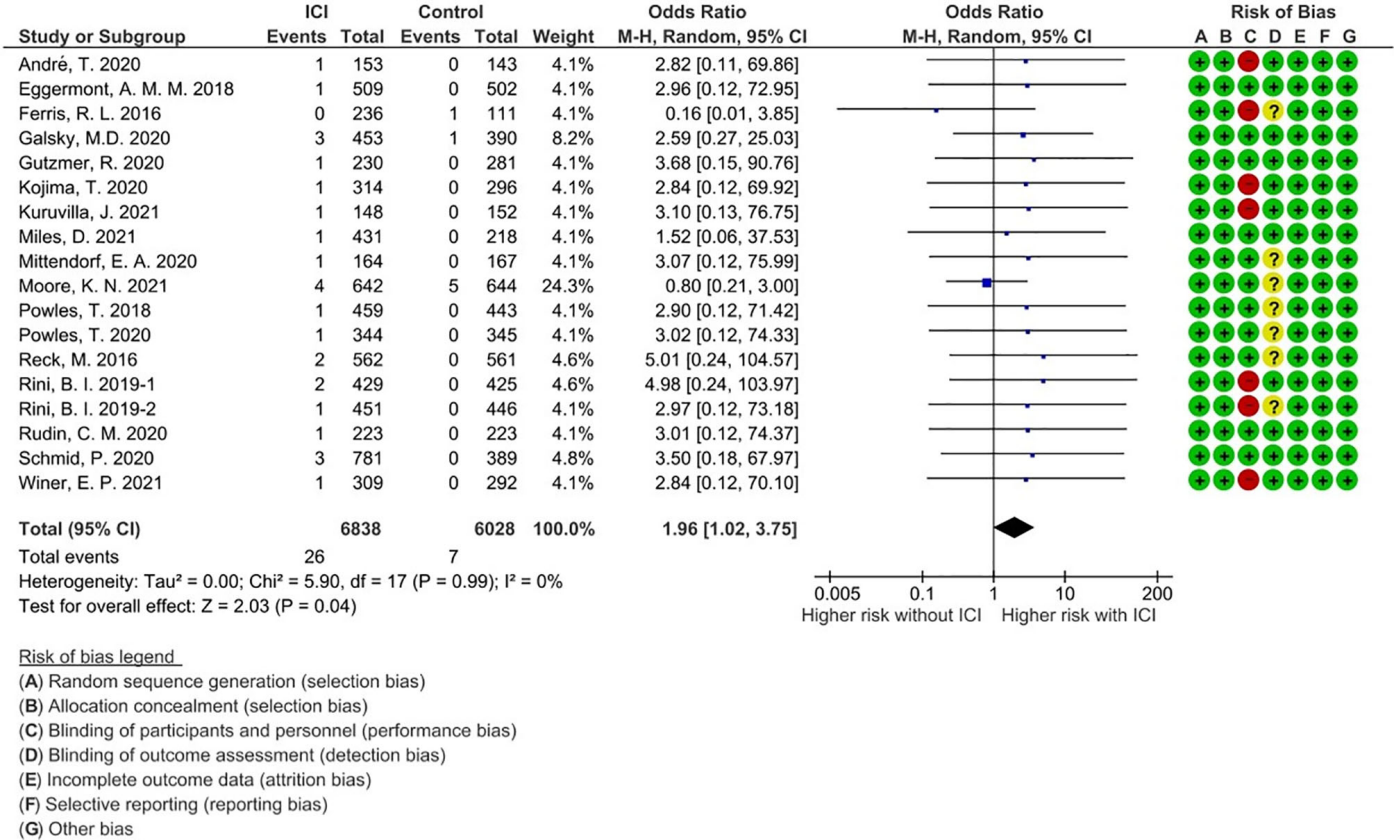
Forest plot with risk of bias summary for incidence of immune checkpoint inhibitor-related myositis.

A total of 33 events of any-grade myositis were observed in 12,866 participants (ICI treatment arm, n = 6,838; non-ICI treatment arm, n = 6,028) from 18 RCTs. Twenty-six events were observed in the ICI arm and seven events were observed in the non-ICI arm. Among the 26 ICI-related myositis, 11 events were caused by anti-PD-1, 13 by anti-programmed death ligand 1 (PD-L1), and two by anti-CTLA-4, suggesting that any ICI can elicit this condition. We calculated OR and 95% CI to compare PD-1/PD-L1 and CTLA-4 blockade therapies. There was no significant difference in frequencies of myositis between two groups (OR 1.07; 95% CI: 0.27-9.41). The overall incidence of ICI-related myositis was 0.38% (26 of 6,838), and did not seem to be affected by the cancer type or ICI regimen. We found that myositis was significantly increased with ICI treatment compared with non-ICI treatment (OR, 1.96; 95% confidence interval [CI] 1.02–3.75; p = 0.04; I^2^ = 0%) (**Figure 2**). These data suggest that ICI-related myositis may be an important component of irAEs in patients receiving ICI therapy.

### Summary of Single Center Analysis

Meta-analysis provides epidemiological data such as the incidence of ICI-related myositis, but it is difficult to analyze detailed clinical characteristics. To understand the prognosis of ICI-related myositis, we first examined our institutional database. A total of 422 patients were identified. Among these, 365 cases were treated with a PD-1 or PD-L1 inhibitor alone, nine were treated with a CTLA-4 inhibitor alone, and 47 cases were treated with a PD-1 inhibitor and CTLA-4 inhibitor combination. There were three cases of ICI-related myositis (incidence 0.71%). Baseline characteristics are shown in **Supplementary Table 1**. All three patients were Asian Japanese and one was female, with mean age 71 ± 6.6 years. Mean treatment duration prior to ICI-related myositis was 13.7 weeks (range 3–34). These patients had muscle weakness with elevated CK with a clinical diagnosis of ICI-related myositis. All were treated with glucocorticoids and other drugs were initiated. However, two patients died (one with pneumonia and one with tumor progression). Patient details are shown in **Supplementary Text 2**.

### Clinical Features and Prognosis of ICI-Related Myositis

To clarify the clinical features of ICI-related myositis in a larger patient population, we evaluated the clinical features of published case reports of ICI-related myositis. Of 75 candidate articles, 63 were identified as eligible (31–93). A further 14 relevant articles (7, 94–106) were added through hand searching. Among the 77 articles, 30 were from Japan, 23 were from USA, five were from France and four were from Canada and Australia (**Supplementary Table 1**). A total of 85 myositis cases associated with at least one ICI were identified. We combined these with the three cases from our institutional database so 88 cases were analyzed in total (precise clinical information of these three patients is noted in the Supplementary Text 2). The most frequent cancer types were lung cancer and melanoma (28 and 27 cases, respectively), followed by urothelial carcinoma. Treatment regimens were as follows: pembrolizumab (anti-PD-1; 37 cases), nivolumab (anti-PD-1; 29 cases), ipilimumab (anti-CTLA-4; four cases), and nivolumab plus ipilimumab (14 cases). Estimated onset time of myositis after ICI administration was 5.6 ± 6.1 weeks and the appearance of autoantibodies varied.

All cases except one were treated with glucocorticoids. Thirty-three patients died, of which 19 deaths were associated with myositis. Because myocarditis is known to be a frequent and fatal complication of ICI-related myositis (107), we compared clinical features of patients with and without myocarditis. Thirty-six of the 88 cases (40.9%) were complicated by myocarditis, confirming the high incidence of myocarditis in ICI-related myositis (**Table 2**).

**Table 2:** Clinical features and prognosis of immune checkpoint inhibitor-related myositis.

		Total	Myocarditis (+)	Myocarditis (-)	*P* value	OR	95% CI
		(n = 88)	(n = 36)	(n = 52)			
Age	Mean, years	68.0	71.9	65.3	0.089		
	Median, years	71	73.5	70.5			
Gender	Male, n	62	27	35	0.484	1.46	0.56-3.77
	Female, n	26	9	17			
Onset date	Mean, weeks	5.6	4.4	6.5	0.462		
	Median, weeks	3.8	3.7	3.9			
Cancer type, (n)		Melanoma (27)	Melanoma (12)	Melanoma (15)	0.075		
		NSCLC (28)	NSCLC (7)	NSCLC (21)			
		UTC (12)	UTC (8)	UTC (4)			
		HNC (3)	HNC (0)	HNC (3)			
		RCC (4)	RCC (2)	RCC (2)			
		HL (2)	HL (0)	HL (2)			
		Others (12)	Others (7)	Others (5)			
ICI, (n)		Nivolumab (29)	Nivolumab (8)	Nivolumab (21)	0.015		
		Pembrolizumab (37)	Pembrolizumab (16)	Pembrolizumab (21)			
		Nivolumab + Ipilimumab (14)	Nivolumab + Ipilimumab (8)	Nivolumab + Ipilimumab (6)			
		Ipilimumab (4)	Ipilimumab (0)	Ipilimumab (4)			
		Others (4)	Others (4)	Others (4)			
Clinical presentation							
	Ptosis, n (%)	45/84 (54)	24/33 (73)	21/51 (41)	0.007	3.81	1.48-9.83
	Ophthalmoplegia, n (%)	28/72 (39)	13/24 (54)	15/48 (31)	0.076	2.60	0.95-7.13
	Dysphasia, n (%)	33/80 (41)	15/31 (48)	18/49 (37)	0.355	1.61	0.65-4.02
	Respiratory muscle paralysis, n (%)	34/82 (41)	21/32 (66)	13/50 (26)	0.001	5.43	2.07-14.26
	Limb weakness, n (%)	79/84 (94)	28/33 (85)	51/51 (100)	0.008	0.05	0.00-0.94
	Rhabdomyolysis, n (%)	8/88 (9)	5/36 (14)	3/52 (6)	0.264	2.63	0.59-11.81
	Myasthenia gravis, n (%)	34/88 (39)	19/36 (53)	15/52 (29)	0.028	2.76	1.13-6.70
	Interstitial lung disease, n (%)	2/88 (2)	0/36 (0)	2/52 (4)	0.511	0.28	0.01-5.94
	Cutaneous manifestations^*1^, n (%)	16/88 (18)	0/36 (0)	16/52 (31)	<0.001	0.03	0.00-0.52
Other irAEs, (n)			Thyroiditis (2)	Hypothyroidism (2)			
			Diarrhea (1)	Thyroiditis (2)			
				Rheumatoid arthritis (1)			
				Colitis (1)			
				Cerebral meningitis (1)			
Autoantibody							
	Anti-AChR, n (%)	17/37 (46)	8/21 (38)	9/16 (56)	0.331	0.48	0.13-1.80
	Anti-striated muscle, n (%)	14/15 (93)	11/12 (92)	3/3 (100)	1.000	1.10	0.04-33.38
	Anti-TIF1γ, n (%)	6/6	0/0	6/6			
Treatment							
	PSL, n (%)	87/88 (99)	36/36 (100)	51/52 (98)	1.000	2.13	0.08-53.67
	IVIG, n (%)	43/88 (49)	19/36 (53)	24/52 (46)	0.665	1.30	0.56-3.06
	Apheresis, n (%)	25/88 (28)	17/36 (47)	8/52 (15)	0.002	4.92	1.81-13.35
	Bio (Infliximab or Rituximab), n (%)	7/88 (8)	6/36 (17)	1/52 (2)	0.017	10.20	1.17-88.84
	Immunosuppressants, (n)		MTX (2), Tac (1), MMF (3)	MTX (3), Tac (3), AZA (1), HCQ (1)			
	Pyridostigmine^*2^, n (%)	12/89 (13)	5/37 (14)	7/52 (13)			
Outcome							
	Recovery of myositis, n (%)	55/81 (68)	12/29 (41)	43/52 (83)	<0.001	0.15	0.05-0.41
Prognosis							
	All deaths, n (%)	33/85 (39)	18/34 (53)	15/51 (29)	0.041	2.70	1.09-6.66
	Directly caused by myositis, n (%)	19/33 (58)	15/18 (83)	4/15 (27)	0.002	13.75	2.54-74.30
	Infection related to myositis, n (%)	4/33 (12)	1/18 (6)	3/15 (20)	0.308	0.24	0.02-2.54
	Progression of a cancer, n (%)	13/33 (39)	3/18 (17)	10/15 (67)	0.005	0.10	0.02-0.52
	Other reasons, n (%)	1/33 (3)	1/18 (6)	0/15 (0)			

NSCLC, non-small cell lung cancer; UTC, urothelial carcinoma; HNC, head and neck cancer; RCC, renal cell carcinoma; HL, Hodgkin’s lymphoma; ICI, immune checkpoint inhibitor; irAEs, immune-related adverse events; AChR, acetylcholine receptor; TIF1γ, transcription Intermediary factor 1γ; PSL, prednisolone; IVIG, intravenous immunoglobulin; Bio, biological therapy; MTX, methotrexate; Tac, tacrolimus; AZA, azathioprine; MMF, mycophenolate mofetil; HCQ, hydroxychloroquine. *1, typical cutaneous signs of dermatomyositis; *2, treatment of myasthenia gravis.

There were some significant differences in clinical characteristics in the group that developed myocarditis. First, we analyzed overall ICI use and found that the occurrence of myocarditis was significantly affected by the type of ICI regimen (p=0.015), but was underpowered to detect an association with a specific regimen. Second, in terms of clinical features at the time of myositis onset, ptosis and respiratory muscle paralysis (type II respiratory failure) were significantly more frequent in the myocarditis group (p=0.007; OR 3.81; 95% CI 1.48–9.83 and p=0.001; OR 5.43; 95% CI 2.07–14.26, respectively). The ORs were calculated from multivariate logistic regression analysis, which also revealed that ptosis and respiratory muscle paralysis were independently associated with the development of myocarditis (p=0.031; OR 2.97; 95% CI 1.10–8.02 and p=0.003; OR 4.54; 95% CI: 1.68–12.23, respectively, **Supplementary Table 2**). Third, in terms of myositis treatment, the myocarditis group received more aggressive treatment such as plasmapheresis and biological agents (p=0.002; OR 4.92; 95% CI 1.81–13.35 and p=0.017; OR 10.20; 95% CI 1.17–88.84, respectively). Finally, in terms of prognosis, myositis symptoms including myocarditis were more refractory to treatment and myositis-related deaths were more common in the group with myocarditis (p=0.002; OR 13.75; 95% CI 2.54–74.30), while the group without myocarditis had a better response to treatment for myositis and more deaths because of cancer progression (p=0.005). These data suggest that myocarditis is a serious complication of ICI-related myositis, the onset of which can be predicted by the development of ptosis.

## Discussion

Because ICI-related myositis is extremely rare, reported only in individual RCTs and case reports, its incidence and clinical phenotype are poorly understood. Here, we provide the first evidence that ICI treatment significantly increases the incidence of myositis compared with non-ICI treatment. Furthermore, detailed analysis of case reports showed that myocarditis with the poorest prognosis had ocular symptoms as the initial manifestation. These results are expected to be useful for the optimization of ICI use in clinical practice.

We show here that ICI-related myositis occurred in 0.38% of patients treated with ICIs, and was significantly more frequent in patients treated with cytotoxic anti-cancer agents. Both anti-PD-1/PD-L1 and anti-CTLA-4 antibodies can cause myositis, but anti-CTLA-4 antibodies have been reported less frequently and may cause myositis less frequently than treatment with anti-PD-1/PD-L1 antibodies. The present study was is underpowered to determine differences in myositis-inducing effects of anti-CTLA-4 and PD-1/PD-L1 antibodies. Further accumulation of cases, pathological verification, and analysis of combination therapy with these two antibodies will be of benefit.

As shown in the previous (7) and current studies, myositis resulting from ICI treatment can be complicated by myasthenia gravis and myocarditis, indicating that ICI-related myositis is a distinct phenotype from IIM (108). Therefore, prognosis in myositis may also differ from that in IIM. Although rapid diagnosis of fatal conditions—especially myocarditis—is necessary, there is currently no standardized method for diagnosing ICI-related myositis. Applying existing diagnostic criteria for IIM and myasthenia gravis to ICI-related diseases may be inappropriate and too late for diagnosis. We identified particular key features, namely ptosis and respiratory paralysis, as important indicators of myocarditis. Based on the current data, we suggest that these patients are closely monitored for myocarditis.

**Figure 3:**
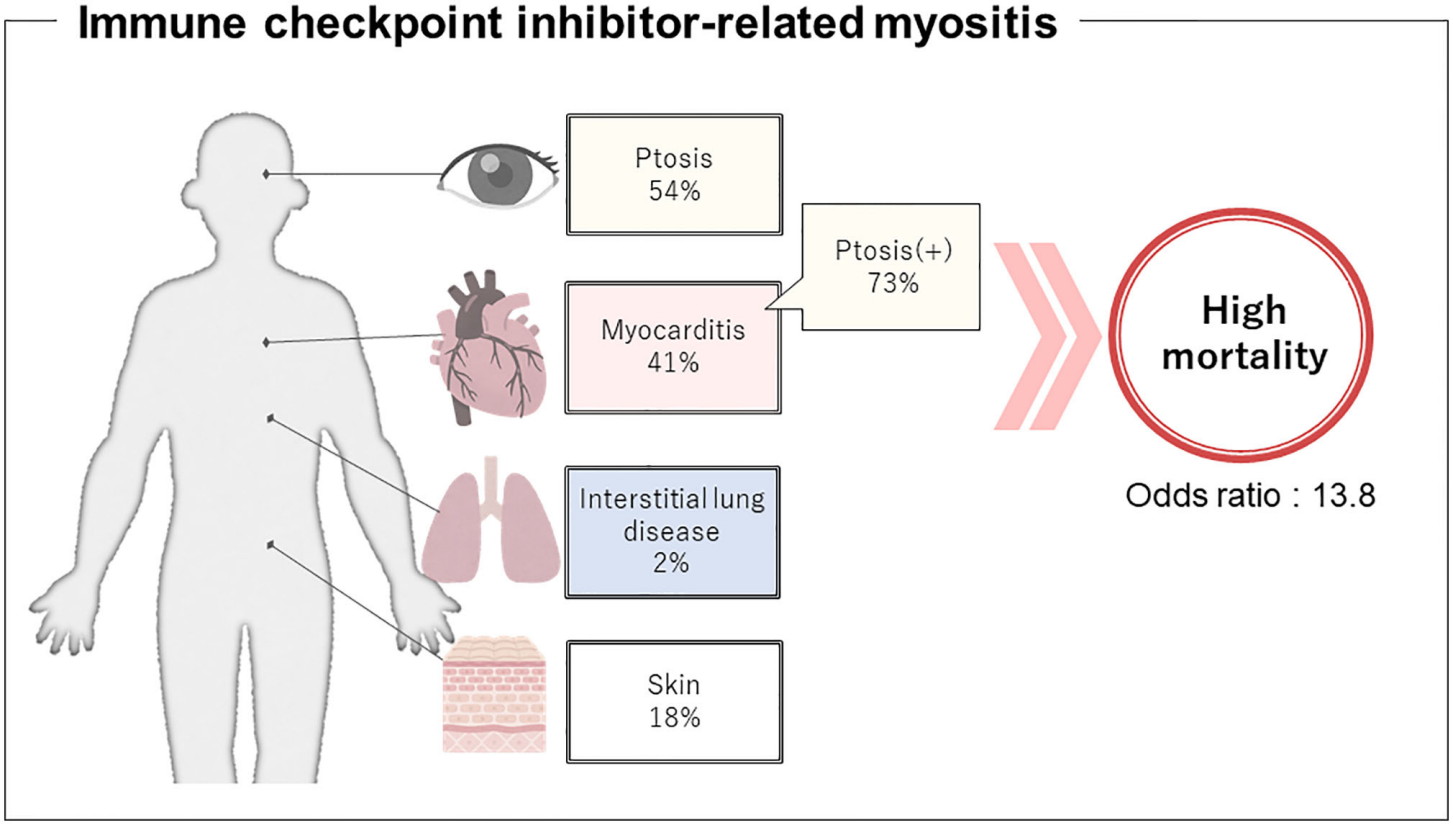
Presumptive clinical features regarding immune checkpoint inhibitor-related myositis. Development of concomitant myocarditis might be predicted by ptosis, therefore, can help prevent the potentially fatal outcome.

ICI myositis may have different clinical features from dermatomyositis and polymyositis as illustrated in **Figure 3**. Typical cutaneous signs of dermatomyositis were seen in 16 cases (18%) and anti-TIF1γ antibody was positive in 6 of them. These cases met the classification criteria for dermatomyositis. There were no cases in which anti-TIF1γ antibody was measured before the start of ICI treatment, and it is unclear whether anti-TIF1γ was induced by ICI-treatment or not. The complication rate of interstitial lung disease was low (2%). Although most cases of ICI-related myositis are negative for myositis-specific antibodies, some cases of positive anti-striated muscle antibodies have been reported (**Supplementary Table 1**), and their diagnostic significance needs to be investigated. The fact that myasthenia gravis without thymoma has been reported to be associated with a specific human leukocyte antigen suggests that there may be some immunological predisposition, although no association between irAEs and human leukocyte antigen has been identified as yet (109, 110). The time from the initiation of ICI to the onset of myositis and characteristic symptoms that overlap with myasthenia gravis, such as ptosis as reported in this study, may be important in distinguishing between ICI-related myositis and myositis because of malignancy.

A limitation of this study is that ICI-related myositis is a rare complication, and while its incidence can be calculated from RCT data, it was not possible to extract the clinical characteristics of individual cases. Diagnosis of myositis and myasthenia gravis or myocarditis is made by clinicians, and there are no standard criteria for diagnosis. In particular, myasthenia gravis is diagnosed even in cases where anti-acetylcholine receptor antibodies are not detected, and further clarification of phenotypes are needed. Myositis-associated autoantibodies can help define subgroups of patients in terms of the clinical phenotype of IIM, but published case reports did not always provide information on myositis-specific antibodies.

Now that this study has shed light on the clinical characteristics of ICI-related myositis, further accumulation of cases from around the world will enable the development of more detailed algorithms for diagnosis and treatment.

## Data Availability Statement

The original contributions presented in the study are included in the article/**Supplementary Material**. Further inquiries can be directed to the corresponding authors.

## Ethics Statement

The studies involving human participants were reviewed and approved by the Ethics committee of Yokohama City University. The patients/participants provided their written informed consent to participate in this study.

## YCU irAE Working Group

The following Authors, who are listed in numerical order, contributed to the work of YCU irAE Working Group: Naoki Hamada, Department of Stem Cell and Immune Regulation, Yokohama City University Graduate School of Medicine, Kanazawa-ku, Japan; Yohei Kirino, Department of Stem Cell and Immune Regulation, Yokohama City University Graduate School of Medicine, Kanazawa-ku, Japan; Motohiko Tokuhisa, Department of Oncology, Yokohama City University Graduate School of Medicine, Kanazawa-ku, Japan; Keiichi Kondo, Department of Urology, Yokohama City University Graduate School of Medicine, Kanazawa-ku, Japan; Noboru Nakaigawa, Department of Urology, Yokohama City University Graduate School of Medicine, Kanazawa-ku, Japan; Nobuaki Kobayashi, Department of Pulmonology, Yokohama City University Graduate School of Medicine, Kanazawa-ku, Japan; Daisuke Sano, Department of Otorhinolaryngology, Head and Neck Surgery, Yokohama City University School of Medicine, Kanazawa-ku, Japan; Maki Hagihara, Department of Stem Cell and Immune Regulation, Yokohama City University Graduate School of Medicine, Kanazawa-ku, Japan; Nobuhiko Oridate, Department of Otorhinolaryngology, Head and Neck Surgery, Yokohama City University School of Medicine, Kanazawa-ku, Japan; Takeshi Kaneko, Department of Pulmonology, Yokohama City University Graduate School of Medicine, Kanazawa-ku, Japan; Yukie Yamaguchi, Department of Environmental Immuno-Dermatology, Yokohama City University Graduate School of Medicine, Kanazawa-ku, Japan; Masahiro Yao, Department of Urology, Yokohama City University Graduate School of Medicine, Kanazawa-ku, Japan; Yasushi Ichikawa, Department of Oncology, Yokohama City University Graduate School of Medicine, Kanazawa-ku, Japan; Hideaki Nakajima, Department of Stem Cell and Immune Regulation, Yokohama City University Graduate School of Medicine, Kanazawa-ku, Japan.

## Author Contributions

NaH, AM, KT-M, and YK designed the research. NaH, AM, KT-M, HoN, and NoH conducted the research, statistical analysis, as well as the interpretation of the data. NaH, AM, KT-M, and YK drafted the manuscript. YS, RY, HoN, and HiN were involved in writing the article or revising it critically for important intellectual content, and all authors approved the final version to be published. NaH, AM and KT-M had full access to all the data in the study and takes responsibility for the integrity of the data and the accuracy of the data analysis.

## Funding

The study was supported from the Yokohama City University Clinical Research Promotion Project. This work was also supported by Yokohama City. This study was supported by Japanese Society for the Promotion of Science Grant-in-Aid for Scientific Research # 21K16289 (NaH).

## Conflict of Interest

The authors declare that the research was conducted in the absence of any commercial or financial relationships that could be construed as a potential conflict of interest.

## Publisher’s Note

All claims expressed in this article are solely those of the authors and do not necessarily represent those of their affiliated organizations, or those of the publisher, the editors and the reviewers. Any product that may be evaluated in this article, or claim that may be made by its manufacturer, is not guaranteed or endorsed by the publisher.
