# Supplementary material for: Incidence and Distinct Features of Immune Checkpoint Inhibitor-Related Myositis From Idiopathic Inflammatory Myositis: A Single-Center Experience With Systematic Literature Review and Meta-Analysis
Source: Front Immunol. 2021 Dec 6;12:803410. doi: 10.3389/fimmu.2021.803410 (PMC8686164; doi:10.3389/fimmu.2021.803410)
Supplement: Supplementary file 1 [file DataSheet_1.docx]

# *Supplementary Material*

# Supplementary Text 1

# *Search formulas*

# PubMed

# #1 Atezolizumab[Title] OR Avelumab[Title] OR Cemiplimab[Title] OR Durvalumab[Title] OR Ipilimumab[Title] OR Nivolumab[Title] OR Pembrolizumab[Title] OR Tremelimumab[Title] OR Spartalizumab[Title] OR Tecentriq[Title] OR Bavencio[Title] OR Libtayo[Title] OR Imfinzi[Title] OR Yervoy[Title] OR Opdivo[Title] OR Keytruda[Title]

# #2 Randomized[Title] OR Randomised[Title] OR RCT[Title] OR Randomly[Title]

# #3 #1 AND #2

# EMBASE

# #1 QUICK SEARCH: Randomized OR Randomised OR RCT OR Randomly

# #2 TITLE: Atezolizumab OR Avelumab OR Cemiplimab OR Durvalumab OR Ipilimumab OR Nivolumab OR Pembrolizumab OR Tremelimumab OR Spartalizumab OR Tecentriq OR Bavencio OR Libtayo OR Imfinzi OR Yervoy OR Opdivo OR Keytruda

# #3 #1 AND #2

# Cochrane Central Register of Controlled Trials

# #1 (Atezolizumab OR Avelumab OR Cemiplimab OR Durvalumab OR Ipilimumab OR Nivolumab OR Pembrolizumab OR Tremelimumab OR Spartalizumab OR Tecentriq OR Bavencio OR Libtayo OR Imfinzi OR Yervoy OR Opdivo OR Keytruda):ti

# #2 (Randomized OR Randomised OR RCT OR Randomly):ti

# #3 #1 AND #2

# Web of Science Core Collection

# #1 TS= (Atezolizumab OR Avelumab OR Cemiplimab OR Durvalumab OR Ipilimumab OR Nivolumab OR Pembrolizumab OR Tremelimumab OR satralizumab OR Tecentriq OR babenco OR liptako OR immirzi OR yerkoy OR optivo OR Keytruda)

# #2 TS=( Randomized OR Randomised OR RCT OR Randomly)

# #3 #1 AND #2

# Supplementary Text 2

# *Case summary*

# Case 1, a 64-year-old man was diagnosed with malignant melanoma of the esophagus after a biopsy of a 3 mm white ridge on upper gastrointestinal endoscopy in April 2020. No metastasis was found, but the patient did not wish to undergo surgery, and combination therapy with nivolumab (80 mg every 3 weeks) and ipilimumab (3 mg/kg every 3 weeks) was started on June 15. Twenty-four days after the first dose, the patient came to our hospital with bilateral ptosis, diplopia, swallowing difficulties, and bilateral lower extremity pain. Physical examination revealed droopy eyelids, difficulty in adduction and supination of both eyes, hoarseness, dysphagia, and muscle weakness in the cervical deltoid muscle. Serum biochemical tests showed elevated CK14857 U/L (normal range, 59-248 U/L). Electromyography showed myogenic changes. Nivolumab and ipilimumab were discontinued for the second time, and the patient was admitted with suspicion of myositis caused by immune checkpoint inhibitor. Autoantibody tests showed slightly positive anti-Acetylcholine receptor antibody (anti-AChR) at 0.3 nmol/L (normal range, <0.2 nmol/L) and slightly positive anti-signal recognition particle antibody (anti-SRP) at 1.1 IU/mL. Anti-nuclear and anti-aminoacyl tRNA synthetase antibodies (anti-ARS) were negative. The patient was diagnosed with complication of myositis and myasthenia gravis. 3 courses of intravenous methylprednisolone therapy (1000 mg/day×3 days) were administered, and CK levels normalized on day 19. Although ptosis, diplopia, and muscle weakness of the neck deltoid improved, dysphagia and hoarseness worsened, and the patient was treated with maintenance therapy with 25 mg/day of prednisolones, 5 plasma exchanges from day 23, and 5 days of intravenous immunoglobulin from day 38. Although a slight improvement in dysphagia was observed, it remained, and the patient was discharged with a gastrostomy. However, in January 2021, he developed aspiration pneumonia and passed away. The cancer was a progressive disease, with worsening liver and lymph node metastasis.

# Case 2, the patient was 72-year-old woman. She was diagnosed with adenoid cystic carcinoma of the submandibular gland after excision of a submandibular gland tumor in August 2016. It was accompanied by multiple lung metastases. In September 2018, she was diagnosed with recurrence of adenoid cystic carcinoma (pT3N0M1) after excision of a 5 mm-sized mass in the right submandibular region. Lung metastasis also worsened, and three cycles of paclitaxel and carboplatin were performed as first line therapy from October. The patient was kept under observation due to prolonged adverse events of myelosuppression. However, in March 2020, it was decided to introduce nivolumab (240 mg every 2 weeks), which was started on April 20, 2020. Twenty-one days after the first dose, the patient came to our hospital with droopy right eyelid and neck myalgia. Physical examination revealed droopy right eyelid, eye movement disorder in both eyes, and muscle weakness in the cervical deltoid, and serum biochemical tests showed elevated CK10404 U/L. MRI showed abnormal high signal in the neck on T2-weighted images. Nivolumab and Ipilimumab were discontinued and the patient was admitted to the hospital with suspicion of myositis/myasthenia gravis due to immune checkpoint inhibitor. The patient's history included a diagnosis of myasthenia gravis of the ocular muscle type in 2012, which had resolved spontaneously with no treatment. Autoantibody tests were positive for anti-Acetylcholine receptor antibody at 22 nmol/L. Anti-nuclear and anti-ARS antibodies were negative. Three courses of intravenous methylprednisolone therapy (1000 mg/day×3 days) were administered, and CK levels normalized on day 44. Ptosis and weakness of the cervical deltoid muscle also improved. Maintenance therapy with 30 mg/day of PSL was given, and PSL was tapered off over the next six months, but there was no relapse of myositis or myasthenia gravis. The anti-Acetylcholine receptor antibody titer decreased to 4.7 nmol/L in August 2020. Since then, the oncology care has been follow-up, and as of June 2021, the patient has been shifted to palliative treatment due to slow increase of lung metastasis.

# Case 3, the patient was 77-year-old man. He became aware of a neck mass in February 2018. A biopsy of the hypopharyngeal mass revealed a diagnosis of squamous cell carcinoma, and he underwent a total pharyngolaryngectomy in April. An image of fluorodeoxyglucose positron emission tomography-computed tomography in June 2019 showed metastases in the hilar region and mediastinum, and he underwent four cycles of carboplatin, 5-FU and cetuximab starting in July. Although maintenance therapy with cetuximab was continued, the patient was diagnosed as progressive disease due to increased mediastinal and hilar lymph node metastasis and appearance of cervical lymph node metastasis in December 2019. He was started nivolumab (240 mg every 2 weeks) in January 2020. The patient continued to receive up to 17 courses by September 2020 without any significant adverse events with cancer complete remission. One week after the last dose of the 17th course, the patient had difficulty walking and eating, and was admitted to our hospital. The patient was unconscious at the time of admission, and it was difficult to assess the exact neurological findings. Serum biochemistry showed elevated CK18642 U/L, and cerebrospinal fluid analysis showed mildly elevated total protein (56 mg/dL) and positive oligoclonal bands. The patient was diagnosed with myositis and meningoencephalitis caused by immune checkpoint inhibitor, and nivolumab was discontinued. Two courses of 3-day intravenous methylprednisolone (1000 mg/day) were administered, and the patient's impaired consciousness rapidly improved. There were some findings of myositis, such as bilateral lower extremity myalgia and symmetrical enhanced areas in the proximal muscles of the lower extremities on T2-weighted MRI images. CK levels normalized on the sixth day and muscle symptoms tended to improve. Autoantibody tests at the time of admission were negative for anti-AChR, anti-nuclear, and anti-ARS antibody. On the 14th day, he had a stroke and became paralyzed in the right upper and lower limbs, and was treated with maintenance therapy with 20 mg/day of PSL for prevention of recurrence of myositis. There was no flare-up of myositis since then, but three months later in December, he passed away due to the progression of cancer.

**Supplementary Figure**

**Figure 1. Funnel plot assessing publication bias** **for odds ratio of immune checkpoint inhibitor-related myositis.**

**
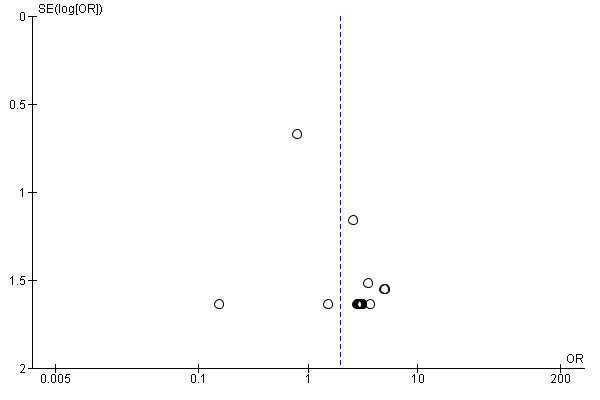
**

**Figure 2. Risk of bias across studies assessed using the Cochrane risk of bias tool.**

**
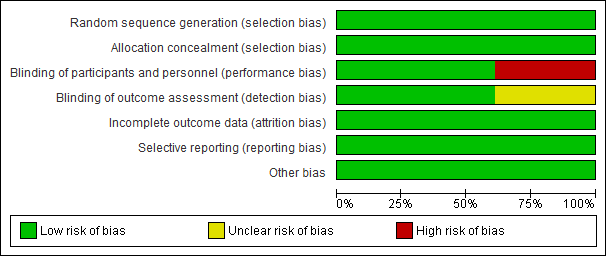
**

**Supplementary Table**

**Table 1. Clinical features and prognosis of immune checkpoint inhibitor-related myositis**

| **Study** | **Country** | **Age, Gender** | **Type of tumor** | **Kind of ICI** | **Ptosis** | **Dysphasia** | **Respiratory muscle paralysis** | **Lim weakness** | **Myo-carditis** | **ILD** | **Skin** | **Other irAEs** | **Auto-antibody** | **Treatment** | **Cause of death** |
| --- | --- | --- | --- | --- | --- | --- | --- | --- | --- | --- | --- | --- | --- | --- | --- |
| Allawh T 2020 | USA | 65years, male | NSCLC | Pemb | - | + | + | + | - | - | - | - |  | PSL, IVIG | Respiratory failure |
| Asano R 2021 | Japan | 15years, male | EBV related naso-pharyngeal cancer | Nivo | - | - | - | + | - | - | + | Hypothiroidism | ANA, TIF1γ | PSL, IVIG, MTX | (Alive) |
| Badovinac S 2018 | Croatia | 64years, female | NSCLC | Nivo | - | - | - | + | - | - | - | Hypothiroidism |  | PSL | (Alive) |
| Behling J 2017 | Germany | 63years, male | Melanoma | Nivo | - | + | - | + | + | - | - | - | ARS, SRP | PSL | Heart failure |
| Berger M 2018 | France | 83years, male | Melanoma | Pemb | - | + | - | + | - | - | + | - | TIF1γ | PSL, IVIG | (Alive) |
| Bilen MA 2016 | USA | 73years, male | UTC | Nivo+Ipi | + | + | + | + | - | - | - | - | anti-SM | PSL, IVIG, PE, IFX | PD of cancer |
| Botta C 2021 | Italy | 72years, male | Bladder cancer | Pemb | + | - | - | + | - | - | - | MG | AChR, ANA | PSL, pyridostigmine | (Alive) |
| Bourgeois-Vionnet J 2018 | France | 79years, male | NSCLC | Nivo | + | + | + | + | - | - | - | - |  | PSL, IVIG | (Alive) |
| Chen JH 2017 | Taiwan | 57years, male | NSCLC | Nivo+Ipi | + | NA | + | + | - | - | - | MG | AChR | PSL, pyridostigmine | Pneumonia, sepsis |
| Claus J 2019 | Belgium | 57years, male | NSCLC | Pemb | - | - | + | + | - | - | - | - |  | PSL | (Alive) |
| Diamantopoulos PT 2017 | Greece | 82years, male | Melanoma | Pemb | + | + | + | + | - | - | - | - |  | PSL, IVIG, PE | Respiratory failure |
| Fazal M 2020 | Australia | 82years, male | Melanoma | Nivo | + | + | + | + | + | - | - | MG | AChR, anti-SM | PSL, IVIG, pyridostigmine | Upper gastrointestinal bleeding |
| Fazel M 2019 | USA | 78years, female | Melanoma | Nivo + Ipi | + | + | + | + | + | - | - | MG | anti-striational antibodies | PSL, IVIG, PE | (Alive) |
| Fox E 2016 | USA | 75years, female | Melanoma | Nivo | - | - | + | + | - | - | - | - |  | PSL | (Alive) |
| Fuentes-Antras J 2020 | Spain | 75years, male | NSCLC | Pemb | + | + | + | + | + | - | - | MG, thyroiditis | AChR,  anti-SM, anti-TPO, Tg, AMA-M2 | PSL, Pyridostigmine, IFX, IVIG | Myocarditis |
| Gandiga PC 2018 | USA | 44years, female | Melanoma | Pemb | - | + | - | + | - | - | - | - |  | PSL, IVIG | PD of cancer |
| Haddox CL 2017 | USA | 78years, male | Melanoma | Pemb | + | + | + | + | + | - | - | - | anti-SM | PSL, PE | Respiratory failure |
| Hamada S 2018 | Japan | 83years, male | NSCLC | Pemb | + | - | - | + | - | - | - | - |  | PSL | (Alive) |
| Hayakawa N 2020 | Japan | 84years, female | UTC | Pemb | + | + | NA | + | - | - | - | MG | anti-titin | PSL, pyridostigmine | (Alive) |
| Hellman JB 2019 | USA | 84years, male | UTC | Pemb | + | + | + | + | + | - | - | - |  | PSL | Respiratory failure |
| Hibino M 2018 | Japan | 83years, male | NSCLC | Pemb | + | - | - | + | - | - | - | MG |  | PSL, pyridostigmine | (Alive) |
| Hinogami H 2019 | Japan | 74years, male | NSCLC | Pemb | - | - | - | + | - | - | + | - | TIF1-γ | PSL, AZA | (Alive) |
| Huh SY 2018 | Korea | 34years, female | Thymic cancer | Pemb | + | + | - | + | - | - | - | MG |  | PSL, IVIG, PE | (Alive) |
| Hunter G 2009 | Canada | 51years, female | Melanoma | Ipi | - | + | - | + | - | - | - | - |  | PSL, IVIG | (Alive) |
| Imai R 2019 | Japan | 70years, male | NSCLC | Pemb | - | NA | NA | + | + | - | - |  |  | PSL, IVIG, HD, Tac | PD of cancer |
| Jeyakumar N 2020 | USA | 86years, male | Cutaneous SCC | Cemiplimab | + | + | + | + | + | - | - | MG | AChR,  anti-SM | PSL, PE, IVIG | Myocarditis, renal failure |
| Johnson DB 2016 | USA | 65years, female | Melanoma | Nivo+Ipi | NA | NA | NA | NA | + | - | - | - |  | PSL | VT |
| Johnson DB 2016 | USA | 63years, male | Melanoma | Nivo+Ipi | NA | NA | NA | NA | + | - | - | - |  | PSL, IFX | Cardiac arrest |
| Kadota H 2019 | Japan | 85years, female | Melanoma | Nivo | - | - | - | + | - | + | - | - | ANA, ARS | PSL | (Alive) |
| Kamo H 2019 | Japan | 78years, male | Urinary cancer | Pemb | + | - | - | + | - | - | - | MG | PM-Scl75, SRP | PSL, PE | PD of cancer |
| Kamo H 2019 | Japan | 72years, female | Lung cancer | Pemb | + | - | - | + | - | - | - | MG |  | PSL | (Alive) |
| Kartolo A 2021 | Canada | 59years, male | NSCLC | Pemb | - | + | - | + | - | - | + | - | ANA | PSL | (Alive) |
| Kim JS 2019 | Korea | 76years, male | NSCLC | Nivo | + | + | + | + | - | - | + | MG | AChR | PSL, IVIG, pyridostigmine | (Alive) |
| Kimura T 2016 | USA | 80years, male | Melanoma | Nivo | + | - | + | + | + | - | - | MG | AChR | PSL, IVIG, PE, IA, pyridostigmine | (Alive) |
| Kobayashi T 2019 | Japan | 25years, male | HL | Nivo | - | - | - | + | - | - | - | - |  | PSL | (Alive) |
| Koh B 2019 | Australia | 61years, male | NSCLC | Nivo | - | + | - | + | - | - | + | - |  | PSL | (Alive) |
| Kosche C 2020 | USA | 63years, male | Melanoma | Nivo | - | - | - | + | - | - | + | NA | ANA | PSL, IVIG | (Alive) |
| Kudo F 2018 | Japan | 42years, male | NSCLC | Nivo | - | - | - | + | - | - | + | - | ANA | PSL | PD of cancer |
| Liao B 2014 | USA | 70years, female | Melanoma | Ipi | + | + | - | + | - | - | - | MG | AChR | PSL, IVIG, PE, pyridostigmine | (Alive) |
| Lie G 2020 | Australia | 79years, male | MPM | Nivo | - | - | - | + | + | - | - | - | ARS | PSL, MMF | NA |
| Lipe DN 2021 | USA | 49years, female | Thymoma | Pemb | - | + | + | + | + | - | - | MG |  | PSL, PE, IVIG | (Alive) |
| Lipe DN 2021 | USA | 67years, male | NSCLC | Durvalumab | + | - | - | + | + | - | - | MG |  | PSL, IVIG, MMF | (Alive) |
| Lipe DN 2021 | USA | 70years, male | UTC | Pemb | + | - | + | - | + | - | - | MG |  | PSL, PE, IFX | Myocarditis |
| Lipe DN 2021 | USA | 81years, female | RCC | Nivo+Ipi | - | - | + | - | + | - | - | MG |  | PSL, PE, IFX, RTX | (Alive) |
| Lipe DN 2021 | USA | 75years, male | Chondroma | Pemb | + | - | - | - | + | - | - | MG | anti-SM | PSL, PE | (Alive) |
| Lipe DN 2021 | USA | 66years, female | RCC | Nivo+Ipi | - | - | + | - | + | - | - | MG |  | PSL, PE, IFX, RTX | (Alive) |
| Lipe DN 2021 | USA | 74years, female | Melanoma | Nivo+Ipi | - | - | + | + | + | - | - | MG | anti-SM | PSL, PE, IVIG | Myocarditis |
| Liu WK 2021 | UK | 71years, male | Melanoma | Nivo | - | - | + | - | + | - | - | - |  | PSL, IVIG, MTX | (Alive) |
| Matsui H 2020 | Japan | 69years, male | UTC | Pemb | + | - | - | + | + | - | - | - |  | PSL, PE | Myocarditis |
| Messer A 2020 | USA | 40years, male | Melanoma | Nivo | - | - | - | + | - | - | + | - | ANA | PSL, IVIG, MTX, HCQ | PD of cancer |
| Monge C 2018 | USA | 79years, male | Prostate cancer | Nivo | NA | - | - | + | + | - | - | - |  | PSL | (Alive) |
| Nakanishi S 2019 | Japan | 78years, male | RCC | Nivo | - | + | + | + | - | - | + | MG | AChR | PSL | Respiratory failure |
| Nasr F 2018 | France | 79years, male | Gastric adenocarcinoma | Pemb | + | + | NA | + | + | - | - |  |  | PSL, IVIG, PE, pyridostigmine, MTX | Myositis, myocarditis |
| Nosaki Y 2020 | Japan | 73years, female | NSCLC | Pemb | + | - | - | + | - | - | - | - |  | PSL | (Alive) |
| Okubo N 2020 | Japan | 57years, male | RCC | Nivo＋Ipi | - | - | - | + | - | - | - | - |  | PSL | (Alive) |
| Onda A 2019 | Japan | 73years, male | NSCLC | Pemb | + | - | - | + | - | - | - | MG | anti-titin | PSL | (Alive) |
| Osaki M 2021 | Japan | 64years, male | NSCLC | Nivo | - | - | + | + | - | - | + | destructive thyroiditis | TIF1γ,  anti-thyroid | PSL, IVIG, Tac | Respiratory failure |
| Pagkopoulou E 2020 | Greece | 64years, male | NSCLC | Pemb | - | - | - | + | - | - | - | RA | RF, ACPA | PSL, MTX | (Alive) |
| Peverelli L 2020 | Italy | 60years, male | NSCLC | Pemb | - | - | - | + | + | - | - | - |  | PSL | Heart failure |
| Rus T 2021 | Slovenia | 63years, male | Melanoma | Nivo | + | + | + | + | - | - | - | - |  | PSL, IVIG, PE | (Alive) |
| Saini L 2017 | Canada | 35years, male | HL | Nivo | - | - | - | + | - | - | - | - |  | PSL | PD of cancer |
| Sanchez-Sancho P 2021 | Spain | 63years, male | Liposarcoma | Pemb | + | + | + | + | + | - | - | MG | AChR | PSL, IVIG, MMF | (Alive) |
| Sessums M 2020 | USA | 74years, male | Bladder cancer | Atezolizumab | + | + | + | + | + | - | - | - |  | PSL | Myocarditis |
| Shah M 2019 | USA | 73years, male | Bladder cancer | Nivo+Ipi | + | NA | + | + | + | - | - | - | anti-SM | PSL, IFX, apheresis, IVIG | PD of cancer |
| Sheik Ali S 2015 | USA | 50years, female | Melanoma | Ipi | - | - | - | + | - | - | + | - | ANA | PSL | (Alive) |
| Shibata C 2019 | Japan | 71years, male | Gastric cancer | Nivo | - | + | - | + | - | - | + | - | TIF1-γ | PSL, IVIG, Tac | PD of cancer |
| Shikano K 2020 | Japan | 81years, male | NSCLC | Nivo | - | - | - | + | - | + | - | - | ARS | PSL, IVIG, Tac | NA |
| Shirai T 2019 | Japan | 83years, male | Melanoma | Pemb | + | + | + | + | + | - | - | MG | ANA, AChR, anti-titin, anti-Kv1.4 | PSL, PE | (Alive) |
| So H 2019 | Japan | 55years, female | Melanoma | Nivo | + | + | + | + | + | - | - | MG | AChR | IVIG, PSL, PE | (Alive) |
| Sutaria R 2019 | USA | 62years, female | Melanoma | Nivo + Ipi | + | - | - | + | - | - | - | MG | AChR | PSL, IVIG | (Alive) |
| Swali R 2020 | USA | 77years, male | Melanoma | Pemb | + | - | + | + | + | - | - | - |  | PSL, IVIG, PE | NA |
| Takahashi S 2020 | Japan | 76years, female | UTC | Pemb | - | - | - | + | + | - | - | - | anti-titin | PSL, IVIG | (Alive) |
| Takatsuki K 2021 | Japan | 71years, female | cancer of unknown primary origin | Pemb | - | - | - | + | - | - | + | Colitis |  | PSL, IVIG | (Alive) |
| Tan RYC 2017 | Singapore | 45years, male | NSCLC | Nivo | + | NA | + | NA | - | - | - | MG | AChR | PSL, IVIG, pyridostigmine | (Alive) |
| Tauber M 2019 | France | 61years, female | Colorectal cancer | Nivo + Ipi | - | - | - | + | - | - | - | - |  | PSL, IVIG | (Alive) |
| Tay SH 2018 | Singapore | 83years, female | UTC | Pemb | + | - | + | + | + | - | - | Thyroiditis | ANA | PSL, IVIG | Pneumonia |
| Todo M 2019 | Japan | 63years, male | Bladder cancer | Pemb | + | - | - | NA | + | - | - | MG, diarrhea, erythema multiforme |  | PSL | (Alive) |
| Uchio N 2020 | Japan | 75years, male | NSCLC | Pemb | NA | NA | NA | + | - | - | - | - |  | - | PD of cancer,  sepsis |
| Valenti-Azcarate R 2020 | Spain | 66years, male | Non-microcytic lung carcinoma | Nivo + Ipi | + | NA | - | + | + | - | - | MG |  | PSL | (Alive) |
| Vallet H 2016 | France | 86years, female | Melanoma | Pemb | + | - | - | + | - | - | - | - |  | PSL, PE | (Alive) |
| Veccia A 2020 | Italy | 65years, male | NSCLC | Nivo | + | + | - | + | + | - | - | MG |  | PSL, IVIG, pyridostigmine | PD of cancer |
| Wiggins CJ 2020 | USA | 74years, male | NSCLC | Pemb | - | - | - | + | - | - | + | - |  | PSL, IVIG | (Alive) |
| Xing Q 2017 | China | 66years, male | NSCLC | Sintilimab | + | + | + | + | + | - | - | MG | AChR | PSL, IVIG, pyridostigmine, PE | (Alive) |
| Yamaguchi Y 2016 | Japan | 70years, female | SCLC | Ipi | - | + | - | + | - | - | + | - | TIF1γ | PSL, IVIG | PD of cancer |
| Yoshioka M 2015 | Japan | 84years, male | Melanoma | Nivo | - | - | + | + | - | - | - | - |  | PSL | (Alive) |

Autoantibodies are indicated only if detected. non small cell lung cancer: NSCLC, Epstein-Barr virus: EBV, urothelial carcinoma: UTC, squamous cell carcinoma: SCC, Hodgkin's lymphoma: HL, malignant pleural mesothelioma: MPM, renal cell carcinoma: RCC, small cell lung cancer: SCLC, immune checkpoint inhibitor: ICI, Pembrolizumab: Pemb, Nivolumab: Nivo, Ipilimumab: Ipi, Interstitial lung disease: ILD, immune-related adverse event: irAE, myasthenia gravis: MG, Rheumatoid arthritis: RA, antinuclear antibody: ANA, anti-transcriptional intermediary factor 1γ antibody: TIF1γ, anti-aminoacyl tRNA synthetase antibody: ARS, anti-signal recognition particle antibody: SRP, anti–striated muscle antibody: anti-SM, anti-acetylcholine receptor antibody: AChR, anti-thyroid peroxidase antibodies: anti-TPO, thyroglobulin: TG, anti-mitochondrial antibody M2: AMA-M2, rheumatoid factor: RF, anti-citrullinated protein/peptide antibody: ACPA, prednisolone: PSL, intravenous immuno-globulin: IVIG, methotrexate: MTX, plasma exchange: PE, infliximab: IFX, azathioprine: AZA, hemodialysis: HD, immune absorption: IA, mycophenolate mofetil: MMF, rituximab: RTX, tacrolimus: Tac, progressive disease: PD.

**Table 2. Age and gender adjusted logistic regression analysis for predicting immune checkpoint inhibitor-related myocarditis**

| Variables | P value | Odds ratio | 95% confidence interval |
| --- | --- | --- | --- |
| Ptosis | 0.031 | 2.972 | 1.101-8.021 |
| Ophthalmoplegia | 0.155 | 2.161 | 0.747-6.253 |
| Dysphasia | 0.376 | 1.535 | 0.594-3.969 |
| Respiratory muscle paralysis | 0.003 | 4.539 | 1.684-12.231 |
